# The radiological features of HPV-positive vs HPV-negative OPSCC at a South African hospital

**DOI:** 10.4102/sajr.v28i1.2976

**Published:** 2024-11-13

**Authors:** Anand Naranbhai, Amir Afrogheh, Suzanne O’Hagan, Johan Grobbelaar, Leon Janse van Rensburg

**Affiliations:** 1 Department of Medical Imaging and Clinical Oncology, Faculty of Medicine and Health Sciences, Stellenbosch University, Cape Town, South Africa; 2 Department of Oral and Maxillofacial Pathology, National Health Laboratory Service and University of the Western Cape, Cape Town, South Africa; 3 Division of Anatomical Pathology, Faculty of Medicine and Health Sciences, Stellenbosch University, Cape Town, South Africa; 4 Department of Surgical Sciences, Faculty of Medicine and Health Sciences, Stellenbosch University, Cape Town, South Africa; 5 Department of Radiology and Diagnostics, Faculty of Dentistry, University of the Western Cape, Cape Town, South Africa

**Keywords:** Radiology, HPV, oropharyngeal squamous carcinoma, South Africa, CT scan, p16 antigen, HPV DNA PCR

## Abstract

**Background:**

Studies have found that, at presentation, human papillomavirus (HPV)-positive oropharyngeal squamous cell carcinoma (OPSCC) has a less advanced primary tumour, more advanced lymph node spread and commonly has cystic metastatic lymph nodes in comparison to HPV-negative OPSCC.

**Objectives:**

To compare the radiological features of HPV-positive and HPV-negative OPSCC in South African patients.

**Method:**

A retrospective cross-sectional study was conducted at a large South African hospital. Eligibility required a histologically proven OPSCC between 2007 and 2023; a p16 antigen test and, if positive, a confirmatory HPV DNA PCR test and a baseline pre-treatment contrast enhanced neck CT scan. All eligible HPV-positive OPSCC patients and a random sample of eligible HPV-negative OPSCC patients were enrolled.

**Results:**

Twenty-one HPV-positive and 55 HPV-negative OPSCC patients were recruited. There was no statistically significant difference in the tumour epicentre location, local advancement (≥ T3 in 67% and 71%, respectively, *p* = 0.54), mean primary tumour size (41 mm vs. 39 mm, *p* = 0.73), lymph node spread (bilateral or more in 67% vs. 82%, *p* = 0.22) or morphologically cystic lymph nodes (10% and 4%, *p* = 0.61).

**Conclusion:**

There was no statistically significant difference in the CT imaging appearances of HPV-positive and HPV-negative OPSCC in the studied sample of South African patients.

**Contribution:**

This study documents the radiological features of OPSCC in a small South African sample population, where HPV-positive and HPV-negative OPSCC could not be distinguished on CT criteria and did not display the classic features described in the literature.

## Introduction

Oropharyngeal squamous cell carcinoma (OPSCC) is a cancer of the epithelium of the oropharynx.^1^ In South Africa, in 2020, approximately 500 new cases were diagnosed and it was the 25th most common cancer.^2^ The anatomic location of OPSCC is complex, with an intermediate prognosis; treatment bears high morbidity and cases are usually already advanced at diagnosis.^3^ These factors contribute to its medical significance.

There are two types of OPSCC.^4,5^ The ‘HPV-negative’ type is primarily caused by tobacco and alcohol exposure and the ‘HPV-positive’ type is caused by human papillomavirus (HPV).^6^ In South Africa, a study of 266 OPSCC patients presenting between 2007 and 2013 found the prevalence of HPV-positive OPSCC to be 5%.^7^ In contrast, in the USA, a study of 8359 OPSCC patients presenting between 2010 and 2011 indicated a prevalence of 65%.^8^ Differences in smoking trends, sexual behaviours (in particular, oral sex) and HPV exposure are believed to be the underlying cause.^9^ Nevertheless, anecdotal evidence suggests HPV-positive OPSCC is increasing in South Africa and so far, there is limited research on OPSCC on the African continent.^7^ Several HPV serotypes cause OPSCC, of which HPV-16 is the most common.^5,7^

The prognosis is favourable for HPV-positive OPSCC compared to HPV-negative OPSCC, and the treatment options vary accordingly.^3,4^ It is therefore important to diagnose which type of cancer is present. Histopathological testing is required for definitive diagnosis.^10,11^ The most utilised test is the p16 antigen immunohistochemistry test. Although not universally applied, evidence suggests that South African patients with a positive P16 antigen test require a more sensitive confirmatory test, such as an HPV DNA PCR test.^7^

Radiological imaging is used for diagnosis, staging and treatment monitoring.^10^ Computed tomography and MRI are the most important modalities used. Several authors have studied if imaging can differentiate between the two types of OPSCC. Three of the most reported features that potentially differentiate between the two types are: HPV-positive OPSCC is less advanced at presentation, has more advanced lymph node spread and more often has morphologically cystic metastatic lymph nodes.^4,12,13,14,15,16^ At present, molecular testing remains the gold standard as none of these features have proven accurate enough to differentiate the two types.

To the authors’ knowledge, there are no studies of the radiological features of OPSCC in Africa. This study aimed to investigate and compare the radiological features of HPV-positive and HPV-negative OPSCC in South African patients, and to determine whether HPV-positive OPSCC in this population exhibits the distinguishing imaging characteristics typically described in the literature.

## Research methods and design

A retrospective, cross-sectional study was conducted. The research population consisted of patients with histologically proven OPSCC between 01 January 2007 and 31 December 2023, identified by searching the National Health Laboratory Service (NHLS) archives at Tygerberg Hospital. The study hospital is a major South African, public, tertiary referral hospital for a population of ±3.4 million people.^17^

**FIGURE 1:**
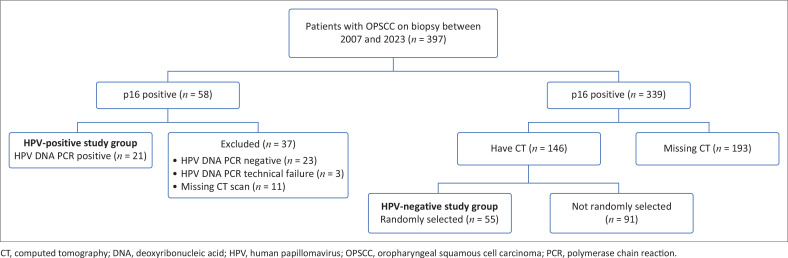
Sample selection process for human papillomavirus-positive and human papillomavirus-negative oropharyngeal squamous carcinoma groups.

Patients with histologically proven OPSCC, a p16 antigen test on the biopsy specimen and available pre-treatment contrast enhanced neck CT scan images on the hospital picture archiving and communications system (PACS), were included. All p16 positive specimens required an HPV DNA PCR test (BD Onclarity^TM^ HPV Assay or Master Diagnostica HPV Direct Flow Chip Assay) to confirm the HPV status. If all three criteria were met, the patients were enrolled into either an HPV-positive group or into an HPV-negative group depending on their HPV status. The HPV-positive group included all eligible HPV-positive cases. The HPV-negative group included a randomly selected subset of the eligible HPV-negative cases. Figure 1 displays the sample selection. The number (*n* = 55) of HPV-negative cases was based on the expectation of 12-16 HPV DNA positive PCR results and the target of four HPV-negative cases for each HPV-positive case. More than expected DNA PCR positive results were received, but only after sample selection was completed, hence the relatively low number of patients in the control group.

Demographic, clinical, radiological and histopathological data were sourced from the hospital medical records department, radiology PACS and the NHLS archives, respectively. Each CT scan was read by two readers (a trainee Diagnostic Radiologist and a board-certified Diagnostic Radiologist) with consensus input provided by a third reader (a board-certified Diagnostic Radiologist). Readers were blinded to the HPV status of the patient, patient identifiers and the previous radiological report. Scans were read according to a standardised template, focussed on only capturing variables of interest to the study.

Continuous variables were analysed using the F-test. Categorical variables were cross-tabulated using the Fisher Exact test. Missing data were excluded. A two-tailed *p*-value less than 0.05 was deemed to be significant. Statistica v14 (TIBCO Software Inc.) was used for all analyses.

### Ethical considerations

An application for full ethical approval was made to the Health Research Ethics Committee of Stellenbosch University, and ethics consent was received on 21 November 2022. The ethics waiver number is S22/10/209; a waiver of informed consent was granted due to the retrospective nature of the study. Data was stored securely and confidentially.

## Results

The final study samples included 21 patients with HPV-positive OPSCC and 55 patients with HPV-negative OPSCC. Demographic and clinical data are presented in Table 1. The mean age of patients was 55 years and 58 years in the HPV-positive and HPV-negative groups respectively. The majority of patients in both groups were male (71% and 78%, *p* = 0.56). Of the 21 HPV-positive cases, HPV-16 was sequenced in 18 patients. HPV-18, HPV-31 and HPV-52 were sequenced in one patient each. HPV-16 and HPV-18 were sequenced in the same patient in one case.

**TABLE 1:** Demographic and clinical features of oropharyngeal squamous cell carcinoma patients according to tumour human papillomavirus status.

Variable	HPV-positive OPSCC			HPV-negative OPSCC			*p*
	*n*	%	s.d.	*n*	%	s.d.	
**Mean age (years)**	55.1	-	9.4	58.1	-	9.2	0.21
**Sex**	-	-	-	-	-	-	0.56
Male	15	71	-	43	78	-	-
Female	6	29	-	12	22	-	-
**Smoking†**	-	-	-	-	-	-	< 0.01*
Yes	10	59	-	37	93	-	-
No	7	41	-	3	8	-	-
Unknown	4	-	-	15	-	-	-
**Alcohol†**	-	-	-	-	-	-	< 0.01*
Yes	6	50	-	27	93	-	-
No	6	50	-	2	7	-	-
Unknown	9	-	-	26	-	-	-
**HIV positive**	-	-	-	-	-	-	1.0*
Yes	1	6	-	5	11	-	-
No	16	94	-	41	89	-	-
Unknown	4	-	-	9	-	-	-
**Currently employed**	-	-	-	-	-	-	0.56*
Yes	7	33	-	13	25	-	-
No	14	67	-	40	75	-	-
Unknown	0	-	-	2	-	-	-

HIV, human immunodeficiency virus; HPV, human papillomavirus; OPSCC, oropharyngeal squamous cell carcinoma; s.d., standard deviation.

* , Excludes unknown.

† , Variably recorded in clinical notes. Timing and amount are unknown.

**FIGURE 2:**
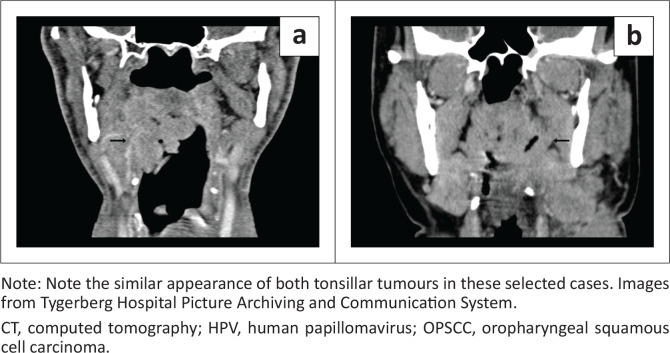
Coronal contrast enhanced CT scan showing a tonsillar tumour in (a) a HPV-positive OPSCC and (b) a HPV-negative OPSCC patient.

**FIGURE 3:**
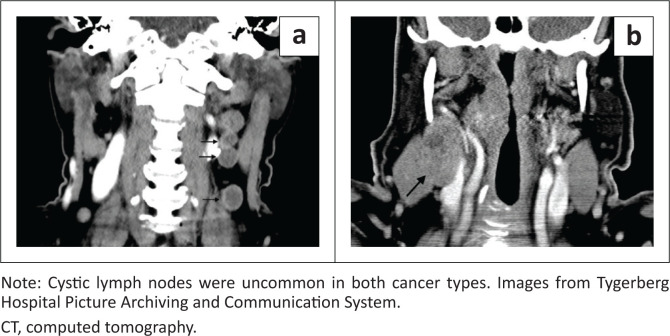
Coronal contrast enhanced neck CT images showing morphologically: (a) cystic and (b) necrotic nodes.

Radiological data are presented in Table 2. In both groups, the palatine tonsils were the most common subsite (52% and 60%) followed by the base of tongue (29% and 18%), with no significant difference between the groups (*p* = 0.35). The size of the primary tumour did not differ between groups (41 mm and 39 mm, *p* = 0.73) (Figure 2). It was equally common to present with a locally advanced tumour (≥ T3 in 67% and 71%, *p* = 0.54). Advanced lymph node spread, defined as bilateral or more disease, was more common in the HPV-negative group but this did not reach statistical significance (67% and 82%, *p* = 0.6). Cystic lymph nodes were seen in two patients in each group and there was no significant difference in morphology of metastatic lymph nodes between groups (10% and 4%, *p* = 0.61) (Figure 3). Rates of distant metastases were similar (18% and 20%, *p* = 1.00).

**TABLE 2:** Radiological findings of oropharyngeal squamous cell carcinoma patients according to human papillomavirus status.

Variable	HPV-positive OPSCC			HPV-negative OPSCC			*p*
	*n*	%	s.d.	*n*	%	s.d.	
**Subsite**	-	-	-	-	-	-	0.35*
Palatine tonsils	11	52	-	33	60	-	-
Base of tongue	6	29	-	10	18	-	-
Other	4	19	-	12	22	-	-
**Mean tumour diameter (mm)**	40.9	-	19	39.2	-	20	0.73
**Radiological T-stage**							
Tx	1	5	-	5	9	-	-
T1	1	5	-	3	5	-	-
T2	5	24	-	8	15	-	-
T3	1	5	-	3	5	-	-
T4	13	62	-	36	65	-	-
T4a	-	-	-	18	-	-	-
T4b	-	-	-	18	-	-	-
**Advanced T-stage†**	-	-	-	-	-	-	0.78
Yes	14	67	-	39	71	-	-
No	7	33	-	16	29	-	-
**Radiological N-stage**							
N0	1	5	-	8	15	-	-
N1	6	29	-	2	4	-	-
N2	10	48	-	38	69	-	-
N2a	-	-	-	0	-	-	-
N2b	-	-	-	17	-	-	-
N2c	-	-	-	21	-	-	-
N3	4	19	-	7	13	-	-
N3a	-	-	-	2	-	-	-
N3b	-	-	-	5	-	-	-
**Advanced N-stage** ‡	-	-	-	-	-	-	0.22
Yes	14	67	-	45	82	-	-
No	7	33	-	10	18	-	-
**Mean lymph node diameter (mm)**	26.4	-	19	20.0	-	18	0.18
**Lymph node morphology**	-	-	-	-	-	-	0.61*
Cystic§	2	10	-	2	4	-	-
Necrotic	12	60	-	33	70	-	-
Solid	6	30	-	12	26	-	-
**Radiological M-stage**	-	-	-	-	-	-	1.0*
M0	14	82	-	39	80	-	-
M1	3	18	-	10	20	-	-
Unknown	4	-	-	6	-	-	-

s.d., standard deviation; HPV, human papillomavirus; OPSCC, oropharyngeal squamous cell carcinoma.

* , Excludes other and/or unknown and/or none.

† , ≥ T3;

‡ , ≥ N2 if HPV-positive, ≥ N2b if HPV-negative;

§ , Enhancing thin (< 2 mm) rim and central homogenous fluid density or intranodal focal homogenous fluid density in which more than 70% of margin is well-defined.

## Discussion

In the evaluated South African study population, HPV-positive and HPV-negative OPSCC could not be differentiated based on CT imaging criteria. Additionally, HPV-positive OPSCC did not exhibit the typical imaging features described in the literature, suggesting that HPV-positive OPSCC in South Africa may present differently compared to other regions. However, the small sample size of this study may limit the generalisability of these findings. To the authors’ knowledge, this is the first study to explore the radiological characteristics of OPSCC on the African continent.

There was no difference in tumour epicentre between the OPSCC types; both favoured the palatine tonsils (52% and 60%, respectively). This is contradictory to Stenmark et al.^8^ who performed a retrospective review of 8359 cases in the USA and found that HPV-positive OPSCC favoured the palatine tonsil but not HPV-negative OPSCC (55% vs. 43%); HPV-negative OPSCC was equally common in the palatine tonsils and base of tongue. The current study finding that the tumour epicentre was located in the palatine tonsils in both types of OPSCC does, however, align with the results of a retrospective review of 476 patients in France conducted by Culie et al.^18^

There was no difference in mean tumour size or in frequency of local advancement between the two groups (67% and 71%). This is contradictory to most studies in the literature.^8,12,13,19,20,21^ In Stenmark et al.’s study, HPV-positive patients tended to present at the T1 or T2 tumour stage significantly more commonly than HPV-negative patients (76% vs. 62%).^8^ An explanation for the difference is that patients in the population of the current study tend to present at late stages of disease because of socio-economic and access related issues. The influence of smoking and alcohol use on the stage at presentation also requires further study.

There was no difference in the frequency of advanced lymph node spread between the two groups (67% and 82%). This is contradictory to most studies in the literature.^8,12,19,20,21^ In Stenmark et al.’s study,^8^ N2 and N3 disease was significantly more common in the HPV-positive group (69% vs. 46%). A small quantum of the difference between this study’s findings and that of Stenmark may be the method of HPV-positivity confirmation. Presumably, Stenmark et al. used P16 testing which has been reported to have an 11% false positivity rate and 8% false-negative rate.^22^ This study used HPV DNA PCR testing which has a different and presumably lower false positive and false-negative rate. Cantrell et al.’s study of 136 patients in the USA did not find a difference between the two groups in advanced lymph node spread.^13^ In their study, each matched pair’s HPV status had been determined by HPV in-situ hybridisation (38 pairs) or HPV16-PCR (30 pairs) testing.

The incidence of cystic metastatic lymph nodes was low in both groups in the current study (10% and 4%), although it was higher in the HPV-positive group. Cantrell et al.’s study found cystic metastatic lymph nodes in 36% and 10% in the HPV-positive and HPV-negative OPSCCs, respectively.^13^ Goldenberg et al. and Morani et al. conducted similar studies and found a pattern similar to Cantrell.^14,15^ Huang et al.’s study reviewed 98 patients in Taiwan using 3T MRI and found that 39% of HPV-positive and 19% of HPV-negative patients had cystic lymph nodes.^12^

The incidence of metastasis in HPV-positive and HPV-negative OPSCC was approximately 18% and 20%, respectively. The most common target organ for metastasis was the lungs (8 of 10 patients in the HPV-positive group and 3 of 3 in the HPV-negative group). The overall incidence of metastasis is higher than in previous studies. Stenmark et al. found an overall rate of metastasis of 3%.^8^ Mirghani et al. found a rate of 1% to 4%.^19^ The current study probably overestimates the rate of lung metastasis. South Africa has a high prevalence of pulmonary tuberculosis and in many cases, the scan readers in this study could not distinguish if the nodules seen were due to pulmonary tuberculosis or metastasis.

Rates of smoking and alcohol use in this HPV-positive study population (59% and 50% respectively) are similar to that reported by Huang et al. (55% and 52% respectively).^12^ The duration and amount of exposure to these substances may differ between the studies and is not available in either. In this study, both HPV-positive and HPV-negative groups had high rates of smoking and alcohol use; however, in the HPV-negative group, nearly all patients had smoking or alcohol related histories. Many participants had missing data for these variables. Nevertheless, the data in this study are in line with the known evidence linking smoking and alcohol use to HPV-negative OPSCC.^6,23^ Regarding HIV status, no significant trend difference in imaging appearance could be found (analysis available on request).

This study has several limitations, the most significant being the small sample size of HPV-positive OPSCC because of the low prevalence in South Africa and the single centre study. Additionally, if upfront HPV DNA PCR results were available, more cases could have been included. However, logistic and financial constraints prevented the acquisition of these results before scan reading took place. A second limitation to this study is that it was retrospective, with limited information on risk factors. A third limitation is the lack of MRI scans because of limited access to MRI. This may have affected the accuracy of assessing radiological variables. Lastly, the assumption was made that the p16 negative patients would also have been HPV DNA PCR negative; however recent studies suggest that a small percentage of p16 negative patients may in fact be HPV-positive (3.8% of patients in a recent study on European and North American patients).^22^

## Conclusion

Human papillomavirus-positive and HPV-negative OPSCC were indistinguishable on CT imaging in this regional cohort of South African patients. Furthermore, HPV-positive OPSCC patients did not display the classic imaging features that have been described in the literature. The need for widespread availability of molecular testing is clearly demonstrated, especially in limited resource settings like Africa, where OPSCC is prevalent, and in a disease like OPSCC, where treatment and prognosis varies based on the results of molecular testing. The results of this initial small study warrant larger prospective studies to determine if these findings may be used in addition to molecular biomarkers to identify the most appropriate treatment options in South African and African populations.
